# Comparative Cost and Utilization Analysis of CAR-T Therapy and Autologous Stem Cell Transplantation for Diffuse Large B-Cell Lymphoma in Germany: Insights from Statutory Health Insurance Billing and Nationwide Inpatient Data

**DOI:** 10.1007/s00432-026-06628-0

**Published:** 2026-09-30

**Authors:** Ann-Cathrine Froitzheim, Melina Sophie Kurte, Katja Gehrke, Sebastian Lempfert, Katja Hesse, Florian Kron

**Affiliations:** 1 Faculty of Medicine, University of Duisburg-Essen, Essen, Germany; 2 VITIS Healthcare Group, Cologne, Germany; 3 Institute for Descriptive Health Data Analysis (OHG), DGDA-Institute, Hamburg, Germany; 4 HCSL Healthcare Consulting, Norderstedt, Germany; 5 Department I of Internal Medicine, Faculty of Medicine and University Hospital Cologne, University of Cologne, Cologne, Germany; 6 FOM University of Applied Sciences, Essen, Germany

**Keywords:** CAR-T, Stem cell transplant, Hematology, Cost, Real world data, Health economics

## Abstract

**Purpose:**

Chimeric Antigen Receptor (CAR) T-cell therapy has transformed the treatment of diffuse large B-cell lymphoma (DLBCL) but carries higher drug acquisition costs than autologous stem cell transplantation (autoSCT), while administration and follow-up costs remain underrepresented in economic evaluations and reimbursement decision-making. This study compares administration-related and subsequent treatment costs of CAR-T and autoSCT for DLBCL from the German statutory health insurance (SHI) perspective.

**Methods:**

A two-step analysis based on two German databases was performed. In step 1, SHI billing data (representing 5 million insured individuals, ≙ 8% of SHI) from 2020–2022 were analyzed longitudinally. Costs were divided into initial regime (IR) (all services except CAR-T drug acquisition) and a following regime (FR). In step 2, a cross-sectional analysis of all German inpatient cases from 2020–2023 (17 million cases/year) assessed inpatient costs.

**Results:**

Step 1 included 12 CAR-T and 59 autoSCT patients. Median IR costs per patient were €61,703 (CAR-T) versus €55,802 (autoSCT), while FR costs were lower for CAR-T (€44,832 versus €69,453). Most frequently reported adverse events (any grade) for CAR-T versus autoSCT were: B-cell aplasia/neutropenia (77% versus 64%), thrombocytopenia (38% versus 64%). Step 2 included 1,229 CAR-T and 2,239 autoSCT inpatient cases (2020–2023). Average LOS was comparable (27.3 versus 27.8 days), and average inpatient costs were lower for CAR-T (€13,991 versus €29,432).

**Conclusion:**

Although associated with higher IR costs, CAR-T therapy in DLBCL demonstrated lower inpatient and FR costs compared to autoSCT. This analysis focused on administration and follow-up costs, excluding drug acquisition costs, characterizing the economic burden beyond drug pricing.

**Supplementary Information:**

The online version contains supplementary material available at https://doi.org/10.1007/s00432-026-06628-0.

## Introduction

Diffuse large B-cell lymphoma (DLBCL) is the most common subtype of non-Hodgkin lymphoma. In Western Europe, the annual number of incident DLBCL cases is projected to rise from approximately 26,000 in 2020 to nearly 28,000 by 2025, reflecting demographic changes and evolving diagnostic practices (Friedberg 2011; Kanas et al. 2022). In Germany, the incidence is estimated at around 7 cases per 100,000 persons (Lenz et al. 2024). Despite high initial response rates to first-line chemotherapy regimes, approximately 30% to 40% of patients relapse or are refractory, requiring further therapeutic intervention (Fabbri et al. 2023).

In relapsed or refractory (r/r) settings, treatment options for transplant-eligible patients include salvage chemotherapy followed by high-dose chemotherapy and consolidation with autologous stem cell transplantation (autoSCT) and, more recently, chimeric antigen receptor T-cell (CAR-T) therapies (axicabtagene ciloleucel and tisagenlecleucel, both approved by the EMA in August 2018), which have been introduced into routine care in Germany since 2018 in the third-line and later (3 L+) setting (Abramson et al. 2024; European Medicines Agency,^ 2026^a, 2026b ; Lenz et al. 2024; Neelapu et al. 2023). Recently, CAR-T therapies have demonstrated superior clinical efficacy compared to autoSCT in populations intended for autoSCT in second-line (2 L) settings (Abramson et al. 2023; Locke et al. 2022). From a health economics perspective, CAR-T therapy has been associated with improved health outcomes, including quality-adjusted life years (QALYs), compared to autoSCT (Choe et al. 2024; Perales et al. 2022). However, these clinical benefits come at the cost of substantial upfront expenditures, particularly related to drug acquisition and manufacturing, resulting in a substantial budget impact and thus a high economic burden for healthcare systems (Skalt et al. 2022).

U.S. cost analyses have shown that the mean total cost of index treatment is significantly higher for CAR-T therapy ($401,693) than for autoSCT ($96,282), yet CAR-T therapy incurs lower non-pharmacy inpatient costs during the index hospitalization (Cui et al. 2022). Whether these findings are transferable to the European healthcare system, especially to Germany’s dual inpatient and outpatient reimbursement framework, is currently unclear.

Despite the growing importance of CAR-T therapies, a comprehensive comparative cost analysis from the German healthcare perspective is lacking. Previous work using statutory health insurance (SHI) claims data in Germany has begun to analyze healthcare utilization patterns (Gehrke et al. 2022; Pacis et al. 2024) or has focused on the entire DLBCL second-line or third-line setting (Borchmann et al. 2023; Moertl et al. 2022). However, comparative cost structure comparisons of CAR-T therapy versus autoSCT, distinguishing between initial treatment and follow-up phases, have not yet been systematically examined.

This study aims to conduct a comparative analysis of inpatient administration and follow-up costs for CAR-T therapy versus autoSCT based on German real-world SHI claim data and hospital billing data. The analysis primarily focuses on healthcare resource utilization (HCRU) and cost dynamics from a German SHI perspective, aiming to supplement existing studies that commonly (merely) focus on acquisition costs and to provide cost inputs relevant for future economic evaluations and reimbursement and pricing decisions for CAR-T and autoSCT in DLBCL.

## Methods

### Study design

**Fig. 1:**
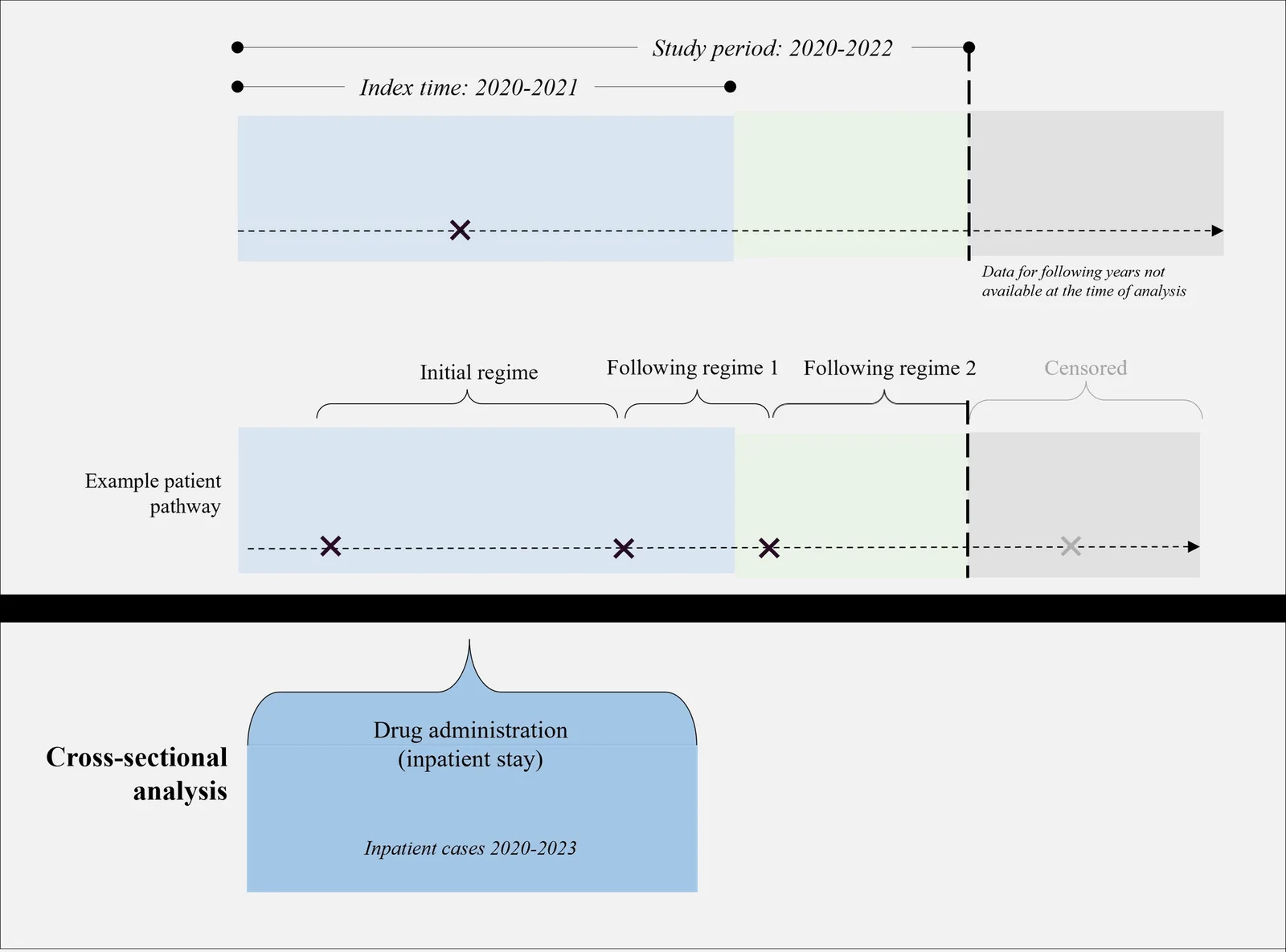
Study approach. Diagram illustrating the study design comprising two analytical components: (1) a cross-sectional analysis of inpatient cases from 2020 to 2023, and (2) a longitudinal analysis tracking individual patients through sequential treatment regimens over time until censoring. Arrows and connecting elements indicate the temporal and analytical relationships between the two components

The retrospective analysis comprised two approaches: (1) a longitudinal analysis based on SHI claim data, covering the initial regime (IR) (CAR-T or autoSCT) and following regimes (FR), and (2) a cross-sectional analysis based on German hospital billing data focusing on the inpatient stay for the administration of the IR. In the context of this analysis, the IR refers to the treatment identified during the index period and does not correspond to the first line (1 L) of therapy. Given the study period, CAR-T treatments detected in the index period most likely reflect the third-line treatment, as earlier/second-line EMA approvals were granted only after the index period. Figure 1 illustrates the combined approach. Since no single data source in Germany captures both the full longitudinal cost trajectory and detailed inpatient resource use simultaneously, a dual approach was applied to ensure comprehensive coverage of all relevant cost dimensions. Analyses were conducted following the guidelines of the German Institute for Quality and Efficiency in Healthcare System valid at time of analysis (IQWiG) (Institut für Qualität und Wirtschaftlichkeit im Gesundheitswesen, 2023).

### Study setting and population

The study population consisted of DLBCL patients in Germany who were treated with either CAR-T therapy or autoSCT as their IR in 2020 or 2021. The analysis reflects the German SHI perspective, covering approximately 90% of the German population. As this is a descriptive, real-world analysis based on aggregated claims and billing data, therefore, no statistical adjustment for potential confounders (e.g., disease severity, age, or follow-up regimen) between the CAR-T and autoSCT groups was performed.

### Longitudinal analysis based on SHI data

Anonymized claims data from the German SHI were evaluated for a longitudinal analysis. The study period was January 1st 2020 until December 31st 2022. At the point of the analysis (November 2024), the latest available complete data year was 2022. The index period was set for 2020 and 2021 to ensure that even cases treated at the end of this period would still have a minimum of one year of follow-up within the study period (which ran through December 2022). The index date was defined as the date of the first documented CAR-T or autoSCT administration within this index period, marking the start of the IR. Follow-up time lasted until the termination of the study period, i.e. end of 2022. Thus, cases were observed for at least one year (Dec 2021-Dec 2022) and a maximum of three years (Jan 2020-Dec 2022). The analysis aimed to follow cases after the initial treatment with either CAR-T or autoSCT. Thus, allocation to a study arm was defined based on the initial treatment (CAR-T arm and autoSCT arm).

For analysis purposes, data were provided in an anonymized and aggregated form. Results based on sample sizes < 5 per arm could not be reported individually due to data protection but are still part of aggregations.

Insured individuals were included if they fulfilled the following eligibility criteria:

- Secured diagnosis of DLBCL in the index period
  - Identified via ICD-code C83.3

- Data availability until the end of the study period (complete data set)
- Treatment of CAR-T and autoSCT in the index period, respectively
  - CAR-T treatment identified via the procedure codes
    - 5-936 (Use of medicinal products for advanced therapies, incl. CAR-T) or
    - 8-802.24 (Transfusion of leukocytes with tumor-specific in vitro preparation, with genetic engineering in vitro preparation, incl. CAR-T, 1-5 subunits) or
    - 8-802.34 (Transfusion of leukocytes with tumor-specific in vitro preparation, with genetic engineering in vitro preparation, incl. CAR-T, more than 5 subunits)
  - autoSCT identified via the procedure codes
    - 5-411.0 (Transplantation of hematopoietic stem cells from the bone marrow, autogenous), or
    - 8-805.0* (Transfusion of peripherally derived hematopoietic stem cells, autogenous), or
    - 8-805.7 (Transfusion of peripherally derived hematopoietic stem cells, retransfusion during the same inpatient stay after failure of the previous transplant), or
    - 8-805.x (Transfusion of peripherally derived hematopoietic stem cells, Other), or
    - 8-805.y (Transfusion of peripherally derived hematopoietic stem cells, not further specified)

Selected cases were analyzed regarding 1) sociodemographic characteristics and comorbidity, 2) FRs, 3) adverse events, 4) HCRU, and 5) costs.

#### Sociodemographic characteristics and comorbidity

The sex and age of the included cases were analyzed. For further sub-analyses, age was grouped into the following categories: 0–59 years, 60–64 years, and 65 years and older.

To assess patients’ comorbidity, two different indices were calculated: an adjusted Charlson Comorbidity Index (CCI) (see below) and the Korean Comorbidity Index (KCI) for predicting survival after stem cell transplant (KCI autoSCT). Both metrics are calculated by evaluating the presence of disease-specific parameters. Indices increase with additional parameters, i.e., higher scores indicate increased comorbidity (Charlson et al. 1987; Park et al. 2021). The CCI was chosen as it remains the most widely used and validated general comorbidity index in claims-based oncology research, allowing comparability with prior studies. Since the eligibility criteria for the analysis defined a DLBCL diagnosis, the prevalence of lymphoma (an item in the CCI) was excluded from the CCI score calculation; therefore, the results must be interpreted in accordance with this adjusted calculation. To address the CCI’s general-purpose limitations in this population, it was deliberately complemented by the disease- and procedure-specific KCI, which was developed specifically to predict survival after stem cell transplantation.

#### Following treatment regimes

Individuals are assigned to either CAR-T or autoSCT regime until another regime is detected. Every two weeks, an assessment is performed to determine whether the assignment of a therapy regime is still valid (“control cycle”).

FRs were divided into six categories, some of which grouped several treatment options: (1) CAR-T; (2) stem cell transplantation; (3) immunochemotherapy, comprising HAP/HAX, EPOCH, and RGDP/GemOX Etoposide; (4) monotherapy, comprising polatuzumab, lenalidomide, brentuximab, pixantrone, ibrutinib, bendamustine, and rituximab; (5) non-defined regime; and (6) censored. Rituximab was only assigned as an independent regime if applied for more than three control cycles (i.e., > 6 weeks). Otherwise, it was assigned to the ongoing previous regime. Since only complete data sets were assessed (i.e., follow-up until the end of the study period possible), all cases were ultimately counted as censored.

Radiotherapy was not assigned as an independent regime but was assigned to the ongoing previous regime. Treatment-free interruptions of the regime of up to six weeks duration (three control cycles) are not to be assessed as treatment discontinuations and do not lead to a change in the regime assignment.

The identified treatment regimes of the insured individual are numbered sequentially. Accordingly, the first identified treatment regime (either CAR-T or autoSCT) in the index quarter is the *(IR)*, followed by the *following regime 1* (FR1), *following regime 2* (FR2), etc.

#### Adverse events

Among possible adverse events, the prevalence of the most frequently occurring events, B-cell aplasia/ neutropenia (ICD-10 codes D70.10-D70.14, D70.18), thrombocytopenia (D69.58), and anemia (D61.10), were analyzed separately (Perales et al. 2022; Appendix Table S3). Adverse events beyond these were grouped and reported as “Other adverse events”. Detailed ICD-10 codes are given in Supplementary Table 1.

#### Healthcare resource utilization

HCRU within the following categories was evaluated, capturing both direct and indirect costs:

- Number of outpatient visits (including outpatient treatment in hospitals).
- Number of inpatient stays and respective length of stay (LOS).
- Number of sick leaves and respective duration.
- Number of drug prescriptions.
- Number of inpatient rehabilitation stays and respective LOS.
- Other (e.g., remedies and aids, transportation).

The duration of regimes was assessed. For standardized HCRU values, a yearly base was calculated by dividing the mean HCRU value by the mean regime duration, which was then multiplied by 365 (days per year).

#### Costs

The following was calculated:

- Costs per utilization category (mean, median, standard derivation (SD), min, max).
- Costs per regime (IR and FRs) (mean, median, SD, min, max).
- Average daily costs per regime, using the following formula:

*Total mean costs across all cost categories [€] / Duration of the respective regime [days]*

Diagnosis information and cost data are independent parameters within the claims data, i.e., it is not possible to clearly distinguish which costs are to be allocated to which diagnosis (e.g., in cases of concomitant diagnoses). Rather, costs are assigned to a regime via the temporal correlation between the documented performance and the detected treatment line. Costs were captured starting from day 0, defined as the first documented service day of the respective treatment regime in the claims data (i.e. the start of the CAR-T or autoSCT regime). All costs attributable to the insured individual are considered disease-related. Actual expenditures were used without adjustment for inflation, as the short observation window and the overlapping calendar periods of both treatment arms make comparable inflation effects likely.

##### Outpatient visits

German outpatient reimbursement is a combination of a lump sum payment and extra-budgetary reimbursement. For the analysis, only extra-budgetary costs were taken into account. Quarterly lump sum payments were not considered since they are paid (from SHI to physicians’ associations) from a pre-defined budget; thus, treating an additional patient does not influence the respective SHI budget (Busse et al. 2017). The cost parameters have further been limited to oncology-related procedures, such as extra fees for the treatment of oncologic patients, imaging procedures, or radiotherapy (see Supplementary Table 2 for the complete list of procedures).

##### Inpatient stays

Inpatient treatment in Germany, including drug acquisition costs during the stay, is reimbursed via diagnosis related group (DRG) flat fees. The entire DRG costs were included in the analysis and associated with the present therapy regime based on the discharge date.

Additional costs are only incurred by pre-defined and negotiated extra fees (“Zusatzentgelte”) and fees for new treatments and diagnostics (“NUB fees”). This is applicable to CAR-T treatment: Additional fees are defined to cover the drug acquisition costs of the available CAR-T products. Since the aim was to identify treatment costs and drug acquisition costs separately, those product costs were deducted from the remaining inpatient costs. However, separating the drug acquisition costs individually for each product was not feasible, as this would require code-based identifiers for each product. Though the required procedure codes for each product have only been available since 2024, i.e., they were not detectable in the database. In addition, product-specific analyses are permitted by the participating SHI companies. In conclusion, the mean drug acquisition costs of all available CAR-T products at the time of the study period were calculated and deducted as a fixed amount.

##### Sick leave payments

Sick leave payments are only applicable to insured individuals who are not retired. Employees in Germany receive sick leave payments from their SHI from the beginning of the seventh week of sick leave, provided that the sick leave is attributed to the same disease (payments for the first six weeks are paid by the employer). A maximum of 78 weeks (within three years; starting from the first sick leave day) of sick leave payment is granted. The daily costs for those payments are summed up for the duration of the sick leave.

##### Drug prescriptions (outpatient)

The actual unit prices paid for the prescripted drugs (Rx) stored in the insured individual’s claims account are used as the costs. These generally correspond to the pharmacy retail price (German: Apothekenverkaufspreis; AVP) after deduction of statutory mandatory discounts. There is no differentiated allocation of pharmaceutical costs according to therapy-relevant drugs.

##### Inpatient rehabilitation

Only inpatient rehabilitation costs covered by SHI (which applies to retired persons) were considered. In contrast, rehabilitation costs for employees are covered by pension funds. Regime allocation was based on the discharge date.

##### Other

Other cost parameters included travelling expenses, as well as remedies and aids. The first documented date of those activities determined to which regime it was allocated.

### Cross-sectional analysis based on German hospital cost data

The cross-sectional analysis was based on real-world inpatient billing data from all German hospitals, obtained through the Data Browser of the Institute for the Remuneration System in Hospitals (InEK), covering the period from 2020 to 2023 (Institut für das Entgeltsystem im Krankenhaus (InEK), 2023). Inpatient cases were identified using the ICD-10-GM diagnosis code for DLBCL, “C83.3,” in combination with the relevant OPS codes for CAR-T therapy (8-802.24 or 8-802.34) or autoSCT (5-411.0-, 8-805.2–5). For data protection reasons, case numbers less than four cases are not disclosed in the InEK data browser (hereinafter referred to as ‘other’).

#### Case characteristics and Healthcare resource utilization

Case characteristics (gender, age) and data on patient clinical complexity level (PCCL) were selected. PCCL is an indicator of resource consumption in hospitals, reflecting the severity of the treatment case. The values range from 0 (no comorbidity or complexity [CC]) to 6 (most severe CC) (Definitionshandbuch 2025 Band 5, InEK GmbH, 2025). HCRU was measured in terms of the average LOS of one hospital stay.

Analytic outcomes were presented as quantities and average parameters of case characteristics and HCRU values over time.

#### Side diagnoses

Side diagnoses were determined based on the operation and procedure (OPS) codes documented during the respective hospital stays for CAR-T therapy or autoSCT administration. Since the OPS codes describe both medical procedures and diseases in the German coding system, secondary diagnoses that do not refer to a respective disorder were excluded. The proportion of side diagnoses in the total number of cases over time was evaluated.

#### Inpatient treatment costs

The inpatient treatment costs for CAR-T therapy and autoSCT were calculated separately for each year from 2020 to 2023. For each year, cost estimations were based on the corresponding national base rate (*Bundesbasisfallwert*) and the specific distribution of DRG observed in that year (GKV-Spitzenverband, 2025). Costs per DRG were derived by multiplying the national base rate of the respective year by the respective case mix index and average LOS associated with each DRG. Total inpatient costs per year were obtained by multiplying the costs per DRG by the distribution (proportion of total cases) of respective cases. Cases allocated to “other” DRGs were distributed evenly across the remaining DRGs. Also, the average inpatient treatment costs across the entire study period were calculated.

The same methodology was applied to nursing care reimbursement, using the average nursing care fee for the respective year (§ 15 Hospital Remuneration Act).

## Results

### Longitudinal analysis based on SHI data

The base population totaled 5,809,782 insured people, of which 2575 had a DLBCL diagnosis. 12 CAR-T patients and 59 autoSCT patients fulfilled all eligibility criteria in the index period.

#### Sociodemographic characteristics and comorbidity

The median age across both arms was 61 (62.5 for CAR-T versus 60.0 for autoSCT). Most patients were female, representing 63% of the overall population, 58% in the CAR-T arm, and 64% in the autoSCT arm. Table 1 shows detailed information.

**Table 1:** [SHI data] Case characteristics

	Total	CAR-T	autoSCT
Age group			
Total [n; %]	71 (100)	12 (100)	59 (100)
0–59 [n; %]	32 (45.07)	4 (33.33)	28 (47.46)
60–64 [n; %]	20 (28.17)	4 (33.33)	16 (27.12)
65+ [n; %]	19 (26.76)	4 (33.33)	15 (25.42)
Median	61.00	62.50	60.00
Mean	56.79	58.67	56.41
SD	12.97	14.70	12.70
Sex			
Female [n; %]	45 (63.38)	7 (58.33)	38 (64.41)
Male [n; %]	26 (36.62)	5 (41.67)	21 (35.59)

In terms of comorbidity, CAR-T and autoSCT patients both had a median (adjusted) CCI of 1 (mean 1.54 versus 1.7) and a median KCI of 0.51 versus 0.64 (mean 0.63 versus 0.73), respectively.

##### Following treatment regimes

In both arms, > 90% of individuals (11/12 in CAR-T arm; 54/59 in autoSCT arm) remained in the initial treatment regime until the end of the study period. In conclusion, one and 5 individuals received one or more FRs). Once an individual transfers to a FR, they are counted as a further case. For instance., the autoSCT arm comprises 59 individuals, of which 5 move to one or more FR(s), thus adding further “regime cases”.

Due to limited population sizes in the FRs, all the following analyses show aggregated results for each study arm, including IR and FRs per arm, but do not indicate the individual regime categories.

#### Adverse events

For both arms, B-cell aplasia/ neutropenia was the most common adverse event, occurring in 77% (10/13) of regime cases in the CAR-T and 64% (42/66) of patients in the autoSCT arm. See Table 2 for detailed adverse events results.

**Table 2:** [SHI data] Adverse events

	CAR-T	autoSCT
B-cell aplasia/neutropenia [*n*; %]	10 (76.92)	42 (63.64)
Thrombocytopenia [n; %]	5 (38.46)	42 (63.64)
Anaemia [n; %]	5 (38.46)	27 (40.91)
Other adverse events [n; %]	12 (92.31)	38 (57.58)

#### Healthcare resource utilization

The median number of inpatient stays was 3 (mean: 2.85) (CAR-T arm) and 3 (mean: 2.95) (autoSCT arm), with a median LOS per stay of 32 (mean 41.85) and 34 (mean: 39.33) days, respectively. On average, patients had 46.23 and 44.12 outpatient visits, in the CAR-T and autoSCT arm, respectively. Sick leave was less frequent and shorter on average in the CAR-T arm (0.54 cases; 68.16 days), compared to the autoSCT arm (0.67; 93.03 days). Drug prescriptions averaged 33.08 (CAR-T arm) and 26.09 (autoSCT arm). More detailed HCRU parameters are provided in Supplementary Table 3.

#### Costs

In the initial CAR-T and autoSCT regimes, inpatient costs average €71,984.58 and €59,511.18, respectively. Adding average costs for FRs of €44,832.13 (CAR-T arm; *n* = 1) and €110,283.73 (autoSCT arm; *n* = 5) total costs per arm amount to €116,816.71 and €169,794.91, respectively. Median inpatient costs were €61,703.93 (CAR-T arm) and €55,802.28 (autoSCT). Median follow-up costs were €44,832.13 (CAR-T arm; *n* = 1) and €69,453.83 (autoSCT; *n* = 5), resulting in total median costs (IR+FR1 + FR2) of €106,536.06 (CAR-T arm) and €125,256.11 (autoSCT arm). An overview of the cost data is provided in Table 3; more SHI cost data are provided in Supplementary Table 4.

**Table 3:** [SHI data] Total costs

		CAR-T		autoSCT	
		Mean	Median	Mean	Median
Drug administration costs [€]	IR costs	71,984.58	61,703.93	59,511.18	55,802.28
Follow-up costs [€]	FR1 costs	44,832.13	44,832.13	52,111.88	29,979.43
	FR2 costs	–	–	58,171.85	39,474.40
Sub-total follow-up		44,832.13	44,832.13	110,283.73	69,453.83
Total		116,816.71	106,536.06	169,794.91	125,256.11

In both groups, inpatient treatment caused the highest costs in the IRs (78% and 79%). In comparison, mean outpatient costs represent < 1% of costs in both IRs. Outpatient drug acquisition costs, which do not include the CAR-T product’s own acquisition costrepresent 6% (autoSCT) and 11% (CAR-T) in the IRs. In the FR1, the primary cost category (regarding mean costs) is inpatient treatment in the autoSCT arm (66%; €34,284.30) and drug acquisition (89%; €40,101.83) in the CAR-T arm.

#### Case characteristics and healthcare resource utilization

Table 4 summarizes case characteristics and HCRU parameters of CAR-T and autoSCT cases between 2020 and 2023.

**Table 4:** [Hospital data] Case characteristics and healthcare utilization

	2020	2021	2022	2023	Total
*Cases*					
CAR-T [n]	276	250	253	450	1229
Female [n; %]	186 (67)	151 (60)	164 (65)	262 (58)	763 (62)
Male [n; %]	90 (33)	99 (40)	89 (35)	188 (42)	466 (38)
autoSCT [n]	606	583	561	489	2239
Female [n; %]	345 (57)	343 (59)	331 (59)	291 (60)	1310 (59)
Male [n; %]	261 (43)	240 (41)	230 (41)	198 (40)	929 (41)
*Age*					
CAR-T [n]	276	250	253	450	1229
≤ 59 [n; %]	118 (43)	78 (31)	104 (41)	182 (40)	482 (39)
60–64 [n; %]	39 (14)	46 (18)	43 (17)	86 (19)	214 (17)
≥ 65 [n; %]	119 (43)	126 (50)	106 (42)	182 (40)	533 (43)
autoSCT [n]	606	583	561	489	2239
≤ 59 [n; %]	253 (42)	245 (42)	253 (45)	195 (40)	946 (42)
60–64 [n; %]	137 (23)	110 (19)	112 (20)	110 (22)	469 (21)
≥ 65 [n; %]	216 (36)	227 (39)	196 (35)	184 (38)	823 (37)
*PCCL*					
CAR-T [n; %]	276	250	253	450	1229
0	36 (13)	25 (10)	39 (15)	63 (14)	163 (13)
1	15 (5)	9 (4)	14 (6)	26 (6)	64 (5)
2	27 (10)	20 (8)	32 (13)	47 (10)	126 (10)
3	56 (20)	63 (25)	82 (32)	136 (30)	337 (27)
4	91 (33)	91 (36)	67 (26)	129 (29)	378 (31)
5	40 (14)	36 (14)	17 (7)	44 (10)	137 (11)
6	11 (4)	6 (2)	2 (1)	5 (1)	24 (2)
autoSCT [n; %]	606	583	561	489	2239
0	5 (1)	2 (0.3)	1 (0.2)	1 (0.2)	9 (0.4)
1	0 (0)	0 (0)	0 (0)	0 (0)	0 (0)
2	0 (0)	1 (0.2)	0 (0)	2 (0.4)	3 (0.1)
3	6 (1)	2 (0.3)	3 (1)	6 (1)	17 (1)
4	124 (20)	140 (24)	133 (24)	114 (23)	511 (23)
5	421 (69)	380 (65)	372 (66)	318 (65)	1491 (67)
6	50 (8)	58 (10)	52 (9)	48 (10)	208 (9)
*Average LOS*					
CAR-T [days (SD)]	29.1 (20.1)	30.3 (25.5)	23.5 (13.3)	26.3 (15.7)	27.3 (18.6)*
autoSCT [days (SD)]	28 (14)	27.9 (14)	27.5 (13.8)	27.6 (15)	27.8 (14.2)*

Over this period, 1229 CAR-T cases were performed, with an increase observed over time (2020: *n* = 276; 2023: *n* = 450). AutoSCT cases totaled 2,239, showing a declining trend during the time horizon (2020: *n* = 606; 2023: *n* = 489).

CAR-T recipients tended to be older than those undergoing autoSCT, with 56% of CAR-T patients < 65 years, compared to 63% in the autoSCT group.

Clinical complexity and hospital resource utilization were measured using the PCCL scoring system. Among CAR-T cases, on average, 58% were assigned PCCL scores of 3 or 4, and 13% PCCL scores 5 or 6 over time. A proportion of CAR-T cases (13%) were also classified as PCCL 0 (no complexity). AutoSCT cases were predominantly assigned higher PCCL scores, with an average of 23% classified as PCCL 4 and 75% as PCCL 5 or 6 over the observation period.

The average LOS for CAR-T cases ranged from 30.3 days (SD 25.5) in 2021 to 23.5 (SD 13.3) in 2022, resulting in an average LOS of 27.3 (SD 18.6) days over the time horizon. The average LOS for autoSCT remained almost stable over time, averaging 27.8 days (SD 14.2).

#### Side diagnoses

Between 2020 and 2023, the most frequently reported side diagnosis among CAR-T cases was cytokine release syndrome, occurring in an average of 78% (range 61–87%). Aplastic anemia and hypokalemia were observed with average rates of 43% (35–51%) and 42% (36–49%), respectively. In autoSCT cases, the most frequent side diagnosis was other secondary thrombocytopenia [88% (87–91%)], followed by aplastic anemia [72% (70–75%)] and hypokalemia [67% (65–69%)]. Detailed information about the prevalence of side diagnoses is presented in Supplementary Table 5.

#### Inpatient treatment costs

**Fig. 2:**
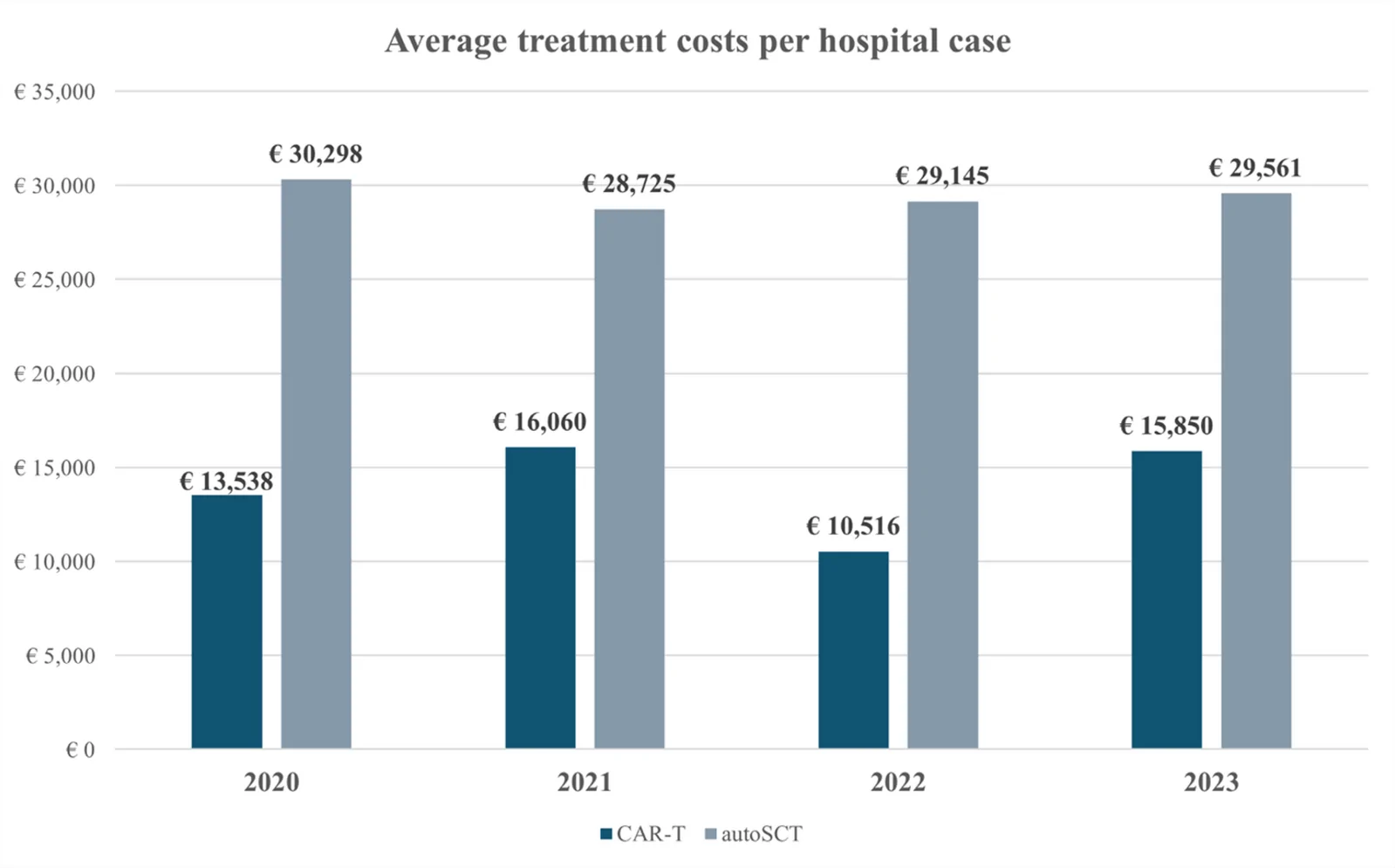
[Hospital data] Average inpatient treatment costs per hospital case 2020–2023. Grouped bar chart comparing average inpatient treatment costs per hospital case in euros for CAR-T and autologous stem cell transplantation (autoSCT) across four years. CAR-T costs were €13,538 (2020), €16,060 (2021), €10,516 (2022), and €15,850 (2023). AutoSCT costs were consistently higher across all years: €30,298 (2020), €28,725 (2021), €29,145 (2022), and €29,561 (2023). The y-axis ranges from €0 to €35,000; the x-axis displays the calendar years 2020 to 2023

Average treatment costs for a CAR-T hospital stay ranged from €10,516 (2022) to €16,060 (2021), with an average of €13,991 over the time horizon (2020–2023) (see Fig. 2).

AutoSCT average treatment costs per hospital case were €29,432 on average over time.

Supplementary Tables 6 and 7 provide the respective underlying DRG distributions for CAR-T and autoSCT administration across all years, while Supplementary Tables 8 and 9 presents detailed cost calculations. CAR-T cases were mainly assigned to DRG R61H (25%) and R61E (22%). The vast majority of autoSCT cases were coded under DRG A15C (96%). An ICU stay was required for approximately 3% of CAR-T cases and 97% of autoSCT cases on average over time. Due to data protection regulations, case numbers below four are not disclosed in the InEK data browser and are reported as “other”. These cases, including those not assignable to a specific DRG (ICU or normal ward), accounted for 8% (CAR-T) and 3% (autoSCT).

## Discussion

### Key findings

This study provides a comparative cost and HCRU analysis of the inpatient and follow-up costs of CAR-T therapy and autoSCT from the perspective of the German SHI system. By leveraging both SHI claims data and hospital billing data, we were able to assess real-world costs and resource use and are typically the primary driver of CAR-T–associated expenses in healthcare systems.

Our results based on SHI data suggest that the IR costs associated with CAR-T therapy were higher than those of autoSCT (median €61,703 versus €55,802). CAR-T therapy was associated with lower follow-up costs (median €44,832 versus €69,453); however, this finding is based on a limited sample and must therefore be interpreted with substantial caution. These findings suggest that while CAR-T results in significantly high upfront drug administration (and acquisition) costs, the economic burden from a long-term SHI perspective may be lower. On both arms, inpatient costs represented the primary cost-driver (> 70%), for both, the IR as well as overall, thus supporting the importance of the cross-sectional analysis of hospital data.

### Context within existing literature

Previous German SHI-based studies have reported increasing costs with advancing treatment lines in DLBCL. Borchmann et al. identified hospitalizations as the dominant cost component, accounting for 71% of total treatment expenditures (Borchmann et al. 2023). Moertl et al. similarly emphasized the economic burden of hospital stays, reporting average total inpatient durations of 44 and 63 days in 2 L and 3 L+ settings (Moertl et al. 2022). Our findings are consistent, confirming that hospitalization remains the primary driver of treatment-related costs beyond drug acquisition costs. Notably, our SHI cohort likely reflects predominantly 3 L+ patients, as the data timeframe terminated before the 2 L EMA approvals in late 2022 (European Medicines Agency, 2026a, 2026b ). The clinical landscape has shifted considerably since the study period (2020–2022/2023). Following the results of the ZUMA-7 trial, which demonstrated superiority of axi-cel over standard care in second-line DLBCL, a shift towards increased CAR-T utilization and reduced autoSCT has been observed. Additionally, the ongoing ZUMA-23 trial is investigating axi-cel as a first-line therapy, which could further shift treatment paradigms. Beyond CAR-T and autoSCT, bispecific antibodies such as glofitamab and epcoritamab have since become additional treatment options for r/r DLBCL, further diversifying the therapeutic landscape beyond the scope of the present analysis (European Medicines Agency, 2025, 2026c ). Consequently, our cost comparisons, reflect an earlier stage of the DLBCL treatment landscape; nonetheless, they remain relevant as a real-world benchmark for the administration and follow-up costs of CAR-T and autoSCT, both of which continue to be used within this evolving landscape. Although the present analysis covers an earlier time frame, the lower follow-up costs observed in CAR-T patients, based on one CAR-T patient with follow-up data, point to a possible, but currently unconfirmed, cost amortization over the patient journey, which would become increasingly relevant as CAR-T moves earlier in the treatment sequence. Future studies based on more recent data are needed to assess whether these cost patterns hold as patient populations and treatment lines shift (Locke et al. 2022; Westin et al. 2023). In contrast to earlier studies, our approach distinguished initial regimes (CAR-T and autoSCT) from follow-up regimes, enabling a more granular understanding of cost developments over time.

### Hospital resource use and clinical characteristics

In this regard, we observed reduced hospital treatment costs for CAR-T therapy administration (€13,991 versus €29,432) and a lower resource consumption in the hospitals compared to autoSCT in our hospital billing dataset. The reduction in hospital-based resource consumption among CAR-T patients is underscored by clinical complexity and treatment intensity values: Although CAR-T patients were slightly older than autoSCT patients (56% <65 years versus 63%), only 3% of CAR-T patients required an ICU stay, compared to 97% in the autoSCT group. The ICU stay was not captured as a separate variable but inferred from the assigned DRG, based on the G-DRG system’s classification of specific DRGs as intensive-care-associated (Supplementary Tables 6 and 7); this reflects the fundamental difference in the underlying DRG assignment. AutoSCT is almost universally billed under the intensive-care-associated DRG A15C, whereas CAR-T is typically billed under the non-intensive-care R61x DRGs, with only a small share of cases classified under a DRG that reflects ICU-level care.

Moreover, more than half (55%) of CAR-T cases were classified with PCCL scores of 0–3, compared to 75% of autoSCT cases classified with PCCL scores of 5–6, indicating lower clinical complexity for CAR-T, which likely contributed to its comparatively lower inpatient treatment costs in this dataset, and pointing to the potential for future outpatient treatment models. PCCL scores are derived from the secondary diagnoses coded during the hospital stay, comprising both pre-existing comorbidities and complications arising during treatment; the higher scores observed for autoSCT could therefore reflect a higher comorbidity burden in that group, a higher rate of coded treatment complications, or both. The available data do not allow us to distinguish between these explanations, and a degree of selection bias cannot be excluded. These patterns align with the growing clinical confidence in CAR-T administration and the shift in treatment guidelines now recommending CAR-T therapy for all 2 L r/r DLBCL patients with early relapse (< 12 months after first-line treatment) or who are primary refractory, which is reflected in the increasing CAR-T and decreasing autoSCT case numbers observed between 2020 and 2023. If CAR-T administration move toward outpatient settings in Germany in the future as recently piloted for cilta-cel in multiple myeloma (Scheid et al. 2026). This could further reduce the inpatient-associated costs that represented the primary cost driver in our; at the same time, it would shift a substantial share of resource use out of the inpatient DRG-based billing captured here, meaning future cost analyses would need to additionally capture outpatient costs to remain representative of the full cost structure.

### Limitations

However, several limitations must be acknowledged. Treatment lines in the sense of the DLBCL treatment guideline (e.g., 2 L vs. 3 L+) were not differentiated in either dataset; based on the valid guidelines in the observation period, most CAR-T therapy cases likely represent 3 L or later treatments. This differential disease burden may have contributed to the observed higher IR costs for CAR-T independently of the treatment modality itself, as later-line patients typically require longer, more complication-prone hospital stays. In addition, the comorbidity indices applied do not capture disease-specific factors such as DLBCL subtype, molecular profile, or overall disease burden, which may influence treatment costs and should be considered when interpreting the results. A subgroup analysis examining whether patients who received autoSCT prior to CAR-T incurred higher upfront costs than those receiving CAR-T as second-line therapy was not feasible with the available data, but would be of interest for future studies. The analysis is descriptive and therefore subject to selection and channeling bias. Differences between the CAR-T and autoSCT patient populations, including disease severity, age, and the follow-up regimes received, were not adjusted for, as the aggregated format and limited sample size of the available data precluded multivariate adjustment or propensity score-based approaches. Additionally, study arm allocation is based on the treatment actually administered; switches between modalities (e.g., due to ineligibility, mobilization failure, or adverse events) cannot be identified in the claims data and are therefore counted under the treatment ultimately received. The claims data do not include a dedicated mortality or discontinuation flag; the exact number of patients who died, changed insurer, or were otherwise lost to follow-up during the study period could therefore not be determined. Due to the aggregated nature of the SHI data further statistical tests (e.g., Mann-Whitney U) could not be applied. The present analysis is best understood as a preliminary, descriptive real-world cost analysis rather than a definitive comparative study. Inpatient treatment costs did not include additional hospital stays for salvage chemotherapy or bridging therapies before autoSCT, respectively, CAR-T therapy if several hospital stays were needed. However, due to the flat-rate nature of the DRG, it is possible that these costs are included if there has only been a single hospitalization. Furthermore, we note that CAR-T administration is associated with a substantial additional fee (approximately €14,875 (Kurte et al. 2023) negotiated individually for each hospital with the German SHI, which may offset some of the observed inpatient costs savings. Since the study focused on the drug administration costs as opposed to drug acquisition costs, the latter were initially subtracted from the identified costs in the SHI analysis. A fixed sum, reflecting the average of available CAR-T products available at the time of the study period, was subtracted. This limitation resulted from the lack of information about the specific product administered in each case (axicabtagene ciloleucel or tisagenlecleucel, the two CAR-T products approved for DLBCL in Germany during the study period). Beyond acquisition costs, this also means we cannot assess whether differences between approved CAR-T products (e.g., in toxicity profile or persistence) contributed to the observed differences in adverse event management, inpatient resource use, or follow-up costs; this would be a valuable focus for future product-level analyses. Although subtracted from the analysis for the above-mentioned reasons, the drug acquisition costs for CAR-T therapy must still be acknowledged. The study findings suggest reduced follow-up costs for CAR-T patients. Thus, the initial acquisition cost may be amortized over the patient journey. Future studies should investigate the amortization potential of (different) CAR-T products related to their potential savings in the follow-up period. Additionally, the representativeness of the participating SHI data (covering approximately 8% of the German SHI-insured population) relative to the overall German SHI population was not formally assessed and cannot be guaranteed, as routine claims data from a subset of statutory health insurance funds may differ from the general SHI population in terms of regional, occupational, or demographic composition. Findings of the SHI analysis are based on rather small population sizes due to ineligibility of cases as well as the limits of the database itself. As a result, aggregation of results was often required to protect data security. Further analyses covering a broader time frame and greater population sizes are required to verify our findings, given the relatively short and temporally lagged SHI data cover only a limited and time-lagged observation period. Recent policy developments in Germany, including expanded access to SHI data via the Forschungsdatenzentrum Gesundheit of BfArm, may enable more robust future analyses with larger samples and longer follow-up periods. The study period also overlapped with the COVID-19 pandemic and related drug-supply constraints. These disruptions may have affected costs through altered procurement prices, treatment delays, additional bridging therapy, repeated assessments, or the use of alternative lymphodepletion regimens. Although such effects may be partially embedded in the observed real-world costs, they could not be quantified separately and may limit the generalizability of the estimates to periods with stable supply chains and hospital capacity.

The most recent version of the DLBCL guideline (Jan 2024) now differentiates between CAR-T eligible and ineligible patients, and includes additional therapy options next to CAR-T and SCT. Future studies should include these updates and evaluate their consequences on resource utilization and costs (Lenz et al. 2024). In particular, bispecific antibodies such as glofitamab and epcoritamab, which represent emerging alternatives to autoSCT and CAR-T therapy, respectively, were not yet approved in Germany during the study period (2020–2022) and could therefore not be included in the present analysis; their impact on costs and healthcare resource utilization should be systematically evaluated in future research (European Medicines Agency,^2025^, ^2026^c).

## Conclusion

In summary, the study results suggest that, in patients with DLBCL, CAR-T therapy, despite its complexity, may lead to lower follow-up treatment costs and reduced resource consumption compared to autoSCT, resulting in a lower demand for inpatient services. These insights could inform future reimbursement and pricing strategies, and may further serve as inputs for economic evaluations, particularly as indications expand and outpatient treatment possibilities evolve. Further research is needed to quantify the long-term budgetary effects of CAR-T therapy in real-world settings.

## Supplementary Information

Below is the link to the electronic supplementary material.

Supplementary Material 1

## Data Availability

No datasets were generated or analysed during the current study.
