# Supplementary material for: Comparative Cost and Utilization Analysis of CAR-T Therapy and Autologous Stem Cell Transplantation for Diffuse Large B-Cell Lymphoma in Germany: Insights from Statutory Health Insurance Billing and Nationwide Inpatient Data
Source: J Cancer Res Clin Oncol. 2026 Sep 30;152(10):190. doi: 10.1007/s00432-026-06628-0 (PMC13631127; doi:10.1007/s00432-026-06628-0)
Supplement: Supplementary file 1 [file 432_2026_6628_MOESM1_ESM.docx]

**APPENDIX**

**Comparative Cost and Utilization Analysis of CAR-T Therapy and Autologous Stem Cell Transplantation for Diffuse Large B-Cell Lymphoma in Germany: Insights from Statutory Health Insurance Billing and Nationwide Inpatient Data**

**Ann-Cathrine Froitzheim, Melina Sophie Kurte, Katja Gehrke, Sebastian Lempfert**, **Katja Hesse, Florian Kron**

Journal of Cancer Research and Clinical Oncology

Corresponding author: Prof Dr. Florian Kron, VITIS Healthcare Group, Cologne, Germany, f.kron@vitis-healthcare.com

Supplementary Table 1: ICD-10-GM Codes of adverse events

Supplementary Table 2: Outpatient fees (and their codes) included in outpatient costs

Supplementary Table 3: [SHI data] Healthcare resource utilization per arm across all regimes

Supplementary Table 4: [SHI data] Costs per category

Supplementary Table 5: [Hospital data] Adverse Events distribution from 2020 – 2023

Supplementary Table 6: DRG distribution of CAR-T cases 2020-2023

Supplementary Table 7: DRG distribution of SCT cases 2020-2023

Supplementary Table 8: Cost Calculation for CAR-T administration (DRG-based)

Supplementary Table 9: Cost Calculation for SCT administration (DRG-based)

Supplementary Table 1: ICD-10-GM Codes of adverse events

| ICD-10-GM Code | Description |
| --- | --- |
| Thrombocytopenia |  |
| D69.58 | Other secondary thrombocytopenia, not classified as transfusion-refractory |
| B-cell aplasia/neutropenia |  |
| D70.10 | Drug-induced agranulocytosis and neutropenia, critical phase less than 4 days |
| D70.11 | Drug-induced agranulocytosis and neutropenia, critical phase 10 to less than 20 days |
| D70.12 | Drug-induced agranulocytosis and neutropenia, critical phase 20 days or more |
| D70.13 | Drug-induced agranulocytosis and neutropenia, critical phase 4 to less than 7 days |
| D70.14 | Drug-induced agranulocytosis and neutropenia, critical phase 7 to less than 10 days |
| D70.18 | Drug-induced agranulocytosis and neutropenia, other clinical courses |
| Anaemia |  |
| D61.10 | Aplastic anemia due to cytostatic therapy |
| Other adverse events |  |
| D76.4 | Cytokine release syndrome |
| E83.38 | Other disorders of phosphorus metabolism and phosphatases |
| E83.58 | Other disorders of calcium metabolism |
| E87.1 | Hypo-osmolality and hyponatremia |
| E87.6 | Hypokalemia |
| F48.0 | Neurasthenia |
| G92.0 | Toxic encephalopathy during immunotherapy |
| G93.3 | Chronic fatigue syndrome |
| I95.2 | Drug-induced hypotension |
| J96.00 | Acute respiratory failure, not elsewhere classified, type I [hypoxemic] |
| J96.01 | Acute respiratory failure, not elsewhere classified, type II [hypercapnic] |
| J96.09 | Acute respiratory failure, not elsewhere classified, unspecified type |
| J96.10 | Chronic respiratory failure, not elsewhere classified, type I [hypoxemic] |
| J96.19 | Chronic respiratory failure, not elsewhere classified, unspecified type |
| R11 | Nausea and vomiting |
| R47.0 | Dysphasia and aphasia |
| R50.2 | Drug-induced fever |
| R50.80 | Fever of unknown origin |
| R50.88 | Other specified fever |

Supplementary Table 2: Outpatient fees (and their codes) included in outpatient costs

| Code | German | English |
| --- | --- | --- |
| 03220 | Zuschlag zur GOP 03000 für die Behandlung und Betreuung eines Patienten mit mindestens einer lebensverändernden chronischen Erkrankung | Surcharge to GOP 03000 for the treatment and care of a patient with at least one life‑changing chronic disease |
| 03221 | Zuschlag zur GOP 03220 für die intensive Behandlung und Betreuung eines Patienten mit mindestens einer lebensverändernden chronischen Erkrankung | Surcharge to GOP 03220 for intensive treatment and care of a patient with at least one life‑changing chronic disease |
| 04220 | Zuschlag zu der Versichertenpauschale nach der Gebührenordnungsposition 04000 für die Behandlung und Betreuung eines Patienten mit mindestens einer lebensverändernden chronischen Erkrankung | Surcharge to the insured flat rate according to GOP 04000 for the treatment and care of a patient with at least one life‑changing chronic disease |
| 04221 | Zuschlag zu der Gebührenordnungsposition 04220 für die intensive Behandlung und Betreuung eines Patienten mit mindestens einer lebensverändernden chronischen Erkrankung | Surcharge to GOP 04220 for intensive treatment and care of a patient with at least one life‑changing chronic disease |
| 04441 | Zusatzpauschale Behandlung einer Systemerkrankung | Additional flat rate for the treatment of a systemic disease |
| 04443 | Zusatzpauschale intensivierte Nachbetreuung nach Tumorbehandlung und/oder Transplantation(en) hämatopoetischer Stammzellen | Additional flat rate for intensified follow‑up care after tumor treatment and/or transplantation(s) of hematopoietic stem cells |
| 15345 | Zusatzpauschale Behandlung und/oder Betreuung eines Patienten mit einer gesicherten onkologischen Erkrankung bei laufender onkologischer Therapie oder Betreuung im Rahmen der Nachsorge | Additional flat rate for treatment and/or care of a patient with a confirmed oncologic disease undergoing ongoing oncologic therapy or follow‑up |
| 25320 | Bestrahlung mit einem Linearbeschleuniger bei bösartigen Erkrankungen – bis 2020 | Radiation with a linear accelerator for malignant diseases – until 2020 |
| 25321 | Bestrahlung mit einem Linearbeschleuniger bei bösartigen Erkrankungen – ab 2021 | Radiation with a linear accelerator for malignant diseases – from 2021 |
| 25322 | Zuschlag zu den Gebührenordnungspositionen 25320 oder 25321 bei Bestrahlung von mehr als 2 Bestrahlungsfeldern | Surcharge to GOP 25320 or 25321 for radiation of more than 2 radiation fields |
| 25323 | Zuschlag zu der Gebührenordnungsposition 25321 bei Bestrahlung in 3-D-Technik (...) | Surcharge to GOP 25321 for 3‑D radiation technique (...) |
| 25340 | Bestrahlungsplanung für die perkutane Bestrahlung | Radiation planning for percutaneous irradiation |
| 25341 | Rechnerunterstützte Bestrahlungsplanung (...)individuelle Dosisplanung | Computer‑assisted radiation planning (...)individualized dose planning |
| 25342 | Rechnerunterstützte Bestrahlungsplanung (...) irreguläre Felder (...) | Computer‑assisted radiation planning (...) irregular fields (...) |
| 34310 | CT‑Untersuchung des Neurocraniums | CT examination of the neurocranium |
| 34311 | CT‑Untersuchung von Teilen der Wirbelsäule | CT examination of parts of the spine |
| 34312 | Zuschlag (...) Serien nach intrathekaler Kontrastmittelgabe | Surcharge (...) series after intrathecal contrast administration |
| 34322 | CT‑Untersuchung der Halsweichteile | CT examination of the neck soft tissues |
| 34340 | CT‑Untersuchung des Oberbauches | CT examination of the upper abdomen |
| 34341 | CT‑Untersuchung des gesamten Abdomens | CT examination of the entire abdomen |
| 34342 | CT‑Untersuchung des Beckens | CT examination of the pelvis |
| 34343 | Zuschlag (...) zweite Serie mit Kontrastmitteln | Surcharge (...) second series with contrast agents |
| 34344 | Zuschlag (...)dynamische Serien | Surcharge (...) dynamic series |
| 34345 | Zuschlag (...) primäre Untersuchung mit Kontrastmittel | Surcharge (...) primary examination with contrast agent |
| 34350 | CT‑Untersuchung der Extremitäten (...) | CT examination of the extremities (...) |
| 34351 | CT‑Untersuchung der Hand, des Fußes (...) | CT examination of the hand, foot (...) |
| 34360 | CT‑gesteuerte Untersuchung (...) Bestrahlungsplanung | CT‑guided examination (...) radiation planning |
| 34410 | MRT‑Untersuchung des Neurocraniums | MRI examination of the neurocranium |
| 34411 | MRT‑Untersuchung von Teilen der Wirbelsäule | MRI examination of parts of the spine |
| 34420 | MRT‑Untersuchung des Gesichtsschädels | MRI examination of the facial skull |
| 34421 | MRT‑Untersuchung der Schädelbasis | MRI examination of the skull base |
| 34422 | MRT‑Untersuchung der Halsweichteile | MRI examination of the neck soft tissues |
| 34440 | MRT‑Untersuchung des Oberbauches | MRI examination of the upper abdomen |
| 34441 | MRT‑Untersuchung des Abdomens | MRI examination of the abdomen |
| 34442 | MRT‑Untersuchung des Beckens | MRI examination of the pelvis |
| 34450 | MRT‑Untersuchung der Extremitäten (...) | MRI examination of the extremities (...) |
| 34451 | MRT‑Untersuchung der Hand, des Fußes (...) | MRI examination of the hand, foot (...) |
| 34452 | Zuschlag (...) mindestens 2 weitere Sequenzen (...)Kontrastmittel | Surcharge (...)at least 2 additional sequences (...)contrast agent |
| 34460 | MRT‑gesteuerte Untersuchung (...) Bestrahlungsplanung | MRI‑guided examination (...) radiation planning |
| 86510 | Behandlung florider Hämoblastosen (...) Onkologie‑Vereinbarung | Treatment of active hematologic malignancies (...) Oncology Agreement |
| 86512 | Behandlung solider Tumore (...) Onkologie‑Vereinbarung | Treatment of solid tumors (...) Oncology Agreement |
| 86514 | Zuschlag (...) intrakavitär applizierte medikamentöse Tumortherapie | Surcharge (...) intracavitary medication‑based tumor therapy |
| 86516 | Zuschlag (...) palliative Versorgung | Surcharge (...) palliative care |
| 86518 | Zuschlag (...) orale medikamentöse Tumortherapie | Surcharge (...) oral medication‑based tumor therapy |

Supplementary Table 3: [SHI data] Healthcare resource utilization per arm across all regimes

|  | CAR-T | autoSCT |
| --- | --- | --- |
| Regime cases [n; %] | 13 (100) | 66 (100) |
| Outpatient visits | | |
| Total | 601 | 2912 |
| Mean | 46.23 | 44.12 |
| Median | 47 | 40.5 |
| SD | 28.74 | 24.93 |
| Min | 10 | 2 |
| Max | 121 | 93 |
| Mean outpatient visits per year | 51.21 | 49.52 |
| Inpatient visits |  |  |
| Total | 37 | 195 |
| Mean | 2.85 | 2.95 |
| Median | 3 | 3 |
| SD | 1.63 | 2.07 |
| Min | 0 | 0 |
| Max | 6 | 12 |
| Mean inpatient visits per year | 3.15 | 3.32 |
| Inpatient days |  |  |
| Total | 544 | 2596 |
| Mean | 41.85 | 39.33 |
| Median | 32 | 34 |
| SD | 41.33 | 25.8 |
| Min | 0 | 0 |
| Max | 169 | 139 |
| Mean inpatient days per year | 46.35 | 44.15 |
| Sick leave cases | | |
| Total | 7 | 44 |
| Mean | 0.54 | 0.67 |
| Median | 0 | 0 |
| SD | 0.66 | 0.93 |
| Min | 0 | 0 |
| Max | 2 | 4 |
| Mean sick leave cases per year | 0.6 | 0.75 |
| Sick leave days | | |
| Total | 886 | 6140 |
| Mean | 68.15 | 93.03 |
| Median | 0 | 0 |
| SD | 100.13 | 125.69 |
| Min | 0 | 0 |
| Max | 257 | 364 |
| Mean sick leave days per year | 75.49 | 104.42 |
| Drug prescriptions | | |
| Total | 430 | 1722 |
| Mean | 33.08 | 26.09 |
| Median | 24 | 21 |
| SD | 19.94 | 17.11 |
| Min | 2 | 2 |
| Max | 67 | 84 |
| Mean number of drug prescription per year | 36.64 | 29.29 |
| Inpatient rehabilitation cases | | |
| Total | 0 | 3 |
| Mean | 0 | 0.05 |
| Median | 0 | 0 |
| SD | 0 | 0.21 |
| Min | 0 | 0 |
| Max | 0 | 1 |
| Mean inpatient rehabilitation cases per year | 0 | 0.05 |
| Duration of regimes (days) | | |
| Total | 4,284 | 21,462 |
| Mean | 329.54 | 325.18 |
| Median | 364 | 364 |
| SD | 90.84 | 92.21 |
| Min | 56 | 42 |
| Max | 364 | 364 |

Supplementary Table 4: [SHI data] Costs per category

|  |  | CAR-T |  |  |  |  |  |  |  | autoSCT |  |  |  |  |  |  |
| --- | --- | --- | --- | --- | --- | --- | --- | --- | --- | --- | --- | --- | --- | --- | --- | --- |
|  | n | **Mean [€]** | **% (Mean)** | **Median [€]** | **% (Median)** | **SD [€]** | **Min.[€]** | **Max.[€]** | n | **Mean [€]** | **% (Mean)** | **Median [€]** | **% (Median)** | **SD [€]** | **Min.[€]** | **Max.[€]** |
| Overall | 13 |  |  |  |  |  |  |  | 66 |  |  |  |  |  |  |  |
| Total |  | 69,895.93 | 100.00 | 61,350.50 | 100.00 | 44,717.21 | 19,195.13 | 197,873.10 |  | 59,001.86 | 100.00 | 54,111.66 | 100.00 | 27,401.58 | 3,527.59 | 144,961.10 |
| Outpatient |  | 595.29 | 0.85 | 160.08 | 0.26 | 880.40 | 0 | 2,300.30 |  | 333.76 | 0.57 | 170.66 | 0.32 | 551.36 | 0 | 4,079.88 |
| Inpatient |  | 52,319.23 | 74.85 | 44,912.87 | 73.21 | 48,170.87 | 0 | 190,975.60 |  | 46,344.67 | 78.55 | 42,531.97 | 78.60 | 24,365.83 | 0 | 128,479.70 |
| Sick leave |  | 5,730.77 | 8.20 | 0 | 0 | 9,779.36 | 0 | 31,986.92 |  | 5,911.07 | 10.02 | 0 | 0 | 9,726.29 | 0 | 45,886.81 |
| Drug acquisition |  | 10,083.54 | 14.43 | 4,919.98 | 8.02 | 11,455.53 | 12.85 | 40,101.83 |  | 4,731.55 | 8.02 | 2,107.45 | 3.89 | 7,605.84 | 106.24 | 41,333.77 |
| Inpatient rehabilitation |  | 0 | 0 | 0 | 0 | 0 | 0 | 0 |  | 387.77 | 0.66 | 0 | 0 | 1,928.59 | 0 | 13,226.94 |
| Other |  | 1,167.10 | 1.67 | 128.36 | 0.21 | 1,994.19 | 0 | 6,706.89 |  | 1,293.04 | 2.19 | 301.50 | 0.56 | 2,553.42 | 0 | 16,638.71 |
| Cost per day |  | 257.55 | 0.37 | 170.49 | 0.28 | 214.41 |  |  |  | 210.31 | 0.36 | 162.72 | 0.30 | 164.36 |  |  |
| Initial regime (IR) | 12 |  |  |  |  |  |  |  | 59 |  |  |  |  |  |  |  |
| Total |  | 71,984.58 | 100.00 | 61,703.93 | 100.00 | 46,038.52 | 19,195.13 | 197,873.10 |  | 59,511.18 | 100.00 | 55,802.28 | 100.00 | 22,472.68 | 29,027.46 | 137,752.60 |
| Outpatient |  | 641.83 | 0.89 | 170.37 | 0.28 | 902.69 | 0 | 2,300.30 |  | 346.35 | 0.58 | 208.46 | 0.37 | 560.56 | 0 | 4,079.88 |
| Inpatient |  | 56,679.16 | 78.74 | 49,071.62 | 79.53 | 47,558.37 | 7,767.99 | 190,975.60 |  | 47,279.67 | 79.45 | 42,904.25 | 76.89 | 16,963.89 | 27,746.28 | 115,616.30 |
| Sick leave |  | 5,817.21 | 8.08 | 0 | 0 | 10,209.02 | 0 | 31,986.92 |  | 6,329.07 | 10.64 | 0 | 0 | 10,109.33 | 0 | 45,886.81 |
| Drug acquisition |  | 7,582.02 | 10.53 | 4,646.55 | 7.53 | 7,376.63 | 12.85 | 20,696.44 |  | 3,710.49 | 6.23 | 1,882.08 | 3.37 | 6,291.98 | 106.24 | 41,333.77 |
| Inpatient rehabilitation |  | 0 | 0 | 0 | 0 | 0 | 0 | 0 |  | 433.78 | 0.73 | 0 | 0 | 2,036.67 | 0 | 13,226.94 |
| Other |  | 1,264.36 | 1.76 | 168.84 | 0.27 | 2,050.41 | 0 | 6,706.89 |  | 1,411.81 | 2.37 | 400.48 | 0.72 | 2,667.93 | 0 | 16,638.71 |
| Cost per day |  | 212.30 | 0.29 | 169.52 | 0.27 | 145.30 |  |  |  | 183.44 | 0.31 | 160.61 | 0.29 | 126.12 |  |  |
| Following regime 1 (FR1) | 1 |  |  |  |  |  |  |  | 4 |  |  |  |  |  |  |  |
| Total |  | 44,832.13 | 100.00 | 44,832.13 | 100.00 | . | 44,832.13 | 44,832.13 |  | 52,111.88 | 100.00 | 29,979.43 | 100.00 | 63,157.59 | 3,527.59 | 144,961.10 |
| Outpatient |  | 36.82 | 0.08 | 36.82 | 0.08 | . | 36.82 | 36.82 |  | 19.99 | 0.04 | 19.97 | 0.07 | 23.08 | 0 | 40.02 |
| Inpatient |  | 0 | 0 | 0 | 0 | . | 0 | 0 |  | 34,284.30 | 65.79 | 4,328.74 | 14.44 | 62,929.43 | 0 | 128,479.70 |
| Sick leave |  | 4,693.48 | 10.47 | 4,693.48 | 10.47 | . | 4,693.48 | 4,693.48 |  | 4,178.83 | 8.02 | 2,427.21 | 8.10 | 5,609.39 | 0 | 11,860.90 |
| Drug acquisition |  | 40,101.83 | 89.45 | 40,101.83 | 89.45 | . | 40,101.83 | 40,101.83 |  | 13,141.70 | 25.22 | 10,798.16 | 36.02 | 12,356.16 | 2,656.24 | 28,314.23 |
| Inpatient rehabilitation |  | 0 | 0 | 0 | 0 | . | 0 | 0 |  | 0 | 0 | 0 | 0 | 0 | 0 | 0 |
| Other |  | 0 | 0 | 0 | 0 | . | 0 | 0 |  | 487.08 | 0.93 | 12.00 | 0.04 | 958.22 | 0 | 1,924.30 |
| Cost per day |  | 800.57 | 1.79 | 800.57 | 1.79 | . |  |  |  | 442.04 | 0.85 | 498.53 | 1.66 | 329.22 |  |  |
| Following regime 2 (FR2) | - |  |  |  |  |  |  |  | 3 |  |  |  |  |  |  |  |
| Total |  | - |  | - |  | - | - | - |  | 58,171.85 | 100.00 | 39,474.40 | 100.00 | 60,580.12 | 9,144.60 | 125,896.60 |
| Outpatient |  | - |  | - |  | - | - | - |  | 504.54 | 0.87 | 182.17 | 0.46 | 721.89 | 0 | 1,331.44 |
| Inpatient |  | - |  | - |  | - | - | - |  | 44,036.83 | 75.70 | 9,219.26 | 23.36 | 68,445.32 | 0 | 122,891.20 |
| Sick leave |  | - |  | - |  | - | - | - |  | 0 | 0 | 0 | 0 | 0 | 0 | 0 |
| Drug acquisition |  | - |  | - |  | - | - | - |  | 13,598.78 | 23.38 | 7,813.16 | 19.79 | 14,588.85 | 2,790.15 | 30,193.04 |
| Inpatient rehabilitation |  | - |  | - |  | - | - | - |  | 0 | 0 | 0 | 0 | 0 | 0 | 0 |
| Other |  | - |  | - |  | - | - | - |  | 31.70 | 0.05 | 33.00 | 0.08 | 31.07 | 0 | 62.10 |
| Cost per day |  |  |  |  |  |  |  |  |  | 429.75 | 0.74 | 562.04 | 1.42 | 230.76 |  |  |

Supplementary Table 5: [Hospital data] Adverse Events distribution from 2020 – 2023

| **Side Diagnosis [% (min-max)]*** | **CAR-T** | **autoSCT** |
| --- | --- | --- |
| Cytokine release syndrome | 78 (61 - 87) | 1 (1 - 2) |
| Aplastic anemia due to cytostatic therapy | 43 (35 - 51) | 72 (70 - 75) |
| Hypokalemia | 42 (36 - 49) | 67 (65 - 69) |
| Toxic encephalopathy under immunotherapy | 33 (26 - 40) | 1 (1 - 1) |
| Other secondary thrombocytopenias, not classified as transfusion-refractory | 29 (23 - 36) | 88 (87 - 91) |
| Drug-induced agranulocytosis and neutropenia: Critical phase 10 days to under 20 days** | 26 (20 - 31) | 25 (21 - 30) |
| Benign essential hypertension: Without specification of a hypertensive crisis | 22 (19 - 26) | 25 (22 - 27) |
| Complications due to drugs or substances | 22 (18 - 27) | 21 (18 - 23) |
| Immunocompromise after radiation therapy, chemotherapy, and other immunosuppressive measures | 21 (20 - 23) | 10 (8 - 12) |
| Drug-induced agranulocytosis and neutropenia: Critical phase 4 days to under 7 days** | 19 (14 - 26) | 20 (17 - 22) |
| Nausea and vomiting | 19 (15 - 24) | 35 (32 - 40) |

* Average percentages across all years

** Staggered values by number of days; total values = CAR-T 84%, autoSCT 97%

Supplementary Table 6: DRG distribution of CAR-T cases 2020-2023

| DRG | 2020 | 2021 | 2022 | 2023 | Average 2020-2023 |
| --- | --- | --- | --- | --- | --- |
| R61H | 26% | 19% | 25% | 29% | 25% |
| R61E | 18% | 18% | 30% | 21% | 22% |
| R61B | 17% | 16% | 14% | 16% | 16% |
| R61A | 13% | 20% | 6% | 7% | 12% |
| R61D | 10% | 6% | 11% | 10% | 9% |
| Other** | 11% | 9% | 7% | 4% | 8% |
| R61F | 0% | 2% | 5% | 5% | 3% |
| A36B* | 4% | 4% | 0% | 2% | 2% |
| R77Z | 0% | 2% | 0% | 2% | 1% |
| A36A* | 0% | 2% | 0% | 1% | 1% |
| R61G | 0% | 0% | 2% | 1% | 1% |
| A15C* | 0% | 0% | 0% | 1% | 0% |

** DRGs for an intensive care unit stay*

*** Due to data protection reasons, case numbers less than four cases (“other”) are not disclosed in the InEK data browser.*

*Abbreviations: CAR-T – Chimeric Antigen Receptor T-Cell; DRG – diagnosis-related groups*

Supplementary Table 7: DRG distribution of SCT cases 2020-2023

| DRG | 2020 | 2021 | 2022 | 2023 | Average 2020-2023 |
| --- | --- | --- | --- | --- | --- |
| A15C* | 96% | 96% | 97% | 94% | 96% |
| Other** | 1% | 3% | 3% | 4% | 3% |
| A13A* | 0% | 1% | 0% | 2% | 1% |
| A11B* | 1% | 0% | 0% | 0% | 0% |
| A09A* | 1% | 0% | 0% | 0% | 0% |

** DRGs for an intensive care unit stay*

*** Due to data protection reasons, case numbers less than four cases (“other”) are not disclosed in the InEK data browser.*

*Abbreviations: DRG – diagnosis-related groups; SCT – autologous stem cell therapy*

Supplementary Table 8: Cost Calculation for CAR-T administration (DRG-based)

| **DRG** | **Cases [n]** | **Cases [%]** | **Average LOS [days]** | **SD LOS [days]** | **First day with additional DRG costs [upper LOS]** | **Case mix average LOS [points]** | **DRG costs average LOS [€]** | **Case mix upper LOS [points]** | **Total days with additional DRG costs upper LOS [days]** | **DRG costs upper LOS [€]** | **Total DRG costs weighted [€]** | **Case mix nursing fee [points]** | **Nursing fees [€]** | **Nursing fees weighted [€]** | **Total costs weighted [€]** |
| --- | --- | --- | --- | --- | --- | --- | --- | --- | --- | --- | --- | --- | --- | --- | --- |
| **2020** |  |  |  |  |  |  |  |  |  |  |  |  |  |  |  |
| R61H | 73 | 26% | 19 | 3.85 | 11 | 0.518 | 1,902 | 0.079 | 9 | 2,594 | 1,189 | 0.863 | 2,710 | 717 | 1,906 |
| R61E | 50 | 18% | 21 | 4.35 | 21 | 1.362 | 5,000 | 0.125 | 1 | 477 | 992 | 0.7367 | 2,570 | 465 | 1,458 |
| R61B | 48 | 17% | 24 | 4.87 | 33 | 2.341 | 8,594 | 0.132 |  | 0 | 1,495 | 1.0072 | 4,059 | 706 | 2,201 |
| R61A | 37 | 13% | 48 | 27.71 | 42 | 3.603 | 13,227 | 0.144 | 7 | 3,886 | 2,294 | 1.1715 | 9,390 | 1,259 | 3,553 |
| R61D | 27 | 10% | 25 | 11.63 | 27 | 1.517 | 5,569 | 0.114 |  | 0 | 545 | 0.9618 | 3,921 | 384 | 928 |
| A36B | 10 | 4% | 54 | 25.38 | 48 | 7.295 | 26,781 | 0.217 | 7 | 5,895 | 1,184 | 2.4104 | 21,737 | 788 | 1,972 |
| Other* | 31 | 11% |  |  |  |  |  |  |  |  | 974 |  |  | 546 | 1,521 |
| **Total** | **276** | **100%** |  |  |  |  |  |  |  |  | **8,673** |  |  | **4,865** | **13,538** |
|  |  |  |  |  |  |  |  |  |  |  |  |  |  |  |  |
| **2021** |  |  |  |  |  |  |  |  |  |  |  |  |  |  |  |
| R61A | 51 | 20% | 37 | 15.33 | 44 | 4.034 | 15,119 | 0.151 |  | 0 | 3,084 | 1.1824 | 7,169 | 1,462 | 4,547 |
| R61H | 48 | 19% | 20 | 3.40 | 12 | 0.535 | 2,005 | 0.079 | 9 | 2,770 | 917 | 0.8354 | 2,773 | 532 | 1,449 |
| R61E | 46 | 18% | 22 | 9.00 | 21 | 1.357 | 5,086 | 0.127 | 2 | 1,149 | 1,147 | 0.7573 | 2,768 | 509 | 1,657 |
| R61B | 41 | 16% | 27 | 9.16 | 33 | 2.372 | 8,890 | 0.132 |  | 0 | 1,458 | 0.98 | 4,261 | 699 | 2,157 |
| R61D | 15 | 6% | 21 | 3.34 | 28 | 1.695 | 6,353 | 0.12 |  | 0 | 381 | 0.9836 | 3,294 | 198 | 579 |
| A36B | 11 | 4% | 35 | 22.12 | 45 | 7.072 | 26,506 | 0.23 |  | 0 | 1,166 | 2.4147 | 13,783 | 606 | 1,773 |
| R77Z | 6 | 2% | 23 | 1.50 | 47 | 4.170 | 15,629 | 0.096 |  | 0 | 375 | 1.1432 | 4,195 | 101 | 476 |
| A36A | 5 | 2% | 69 | 28.69 | 57 | 11.897 | 44,590 | 0.281 | 13 | 13,481 | 1,161 | 2.9114 | 32,668 | 653 | 1,815 |
| R61F | 5 | 2% | 19 | 1.96 | 15 | 1.003 | 3,759 | 0.138 | 5 | 2,379 | 123 | 1.2029 | 3,649 | 73 | 196 |
| Other* | 22 | 9% |  |  |  |  |  |  |  |  | 947 |  |  | 466 | 1,413 |
| **Total** | **250** | **100%** |  |  |  |  |  |  |  |  | **10,760** |  |  | **5,301** | **16,060** |
|  |  |  |  |  |  |  |  |  |  |  |  |  |  |  |  |
| **2022** |  |  |  |  |  |  |  |  |  |  |  |  |  |  |  |
| R61E | 77 | 30% | 21 | 3.58 | 21 | 1.345 | 5,155 | 0.126 | 1 | 483 | 1,716 | 0.7377 | 2,755 | 838 | 2,555 |
| R61H | 62 | 25% | 19 | 2.46 | 12 | 0.531 | 2,035 | 0.079 | 8 | 2,418 | 1,091 | 0.8157 | 2,811 | 689 | 1,780 |
| R61B | 35 | 14% | 24 | 5.80 | 36 | 2.584 | 9,905 | 0.132 |  | 0 | 1,370 | 1.0049 | 4,378 | 606 | 1,976 |
| R61D | 28 | 11% | 22 | 4.38 | 28 | 1.742 | 6,677 | 0.121 |  | 0 | 739 | 0.9418 | 3,676 | 407 | 1,146 |
| R61A | 16 | 6% | 36 | 13.34 | 46 | 4.231 | 16,218 | 0.147 |  | 0 | 1,026 | 1.1619 | 7,633 | 483 | 1,508 |
| R61F | 12 | 5% | 21 | 2.66 | 14 | 0.975 | 3,737 | 0.138 | 8 | 4,452 | 388 | 1.1762 | 4,573 | 217 | 605 |
| R61G | 5 | 2% | 22 | 4.22 | 16 | 0.933 | 3,576 | 0.135 | 7 | 3,415 | 138 | 0.7641 | 2,996 | 59 | 197 |
| Other* | 18 | 7% |  |  |  |  |  |  |  |  | 495 |  |  | 253 | 748 |
| **Total** | **253** | **100%** |  |  |  |  |  |  |  |  | **6,964** |  |  | **3,552** | **10,516** |
|  |  |  |  |  |  |  |  |  |  |  |  |  |  |  |  |
| **2023** |  |  |  |  |  |  |  |  |  |  |  |  |  |  |  |
| R61H | 132 | 29% | 20 | 5.05 | 12 | 0.55 | 2,200 | 0.078 | 9 | 2,669 | 1,428 | 0.8294 | 3,730 | 1,094 | 2,522 |
| R61E | 93 | 21% | 21 | 4.15 | 21 | 1.335 | 5,341 | 0.123 | 1 | 492 | 1,205 | 0.7429 | 3,526 | 729 | 1,934 |
| R61B | 74 | 16% | 27 | 9.75 | 37 | 2.914 | 11,658 | 0.14 |  | 0 | 1,917 | 0.9897 | 6,094 | 1,002 | 2,919 |
| R61D | 45 | 10% | 22 | 6.56 | 28 | 1.788 | 7,153 | 0.12 |  | 0 | 715 | 0.9389 | 4,818 | 482 | 1,197 |
| R61A | 33 | 7% | 47 | 20.16 | 50 | 5.178 | 20,716 | 0.151 |  | 0 | 1,519 | 1.2553 | 13,465 | 987 | 2,507 |
| R61F | 21 | 5% | 21 | 6.22 | 17 | 1.036 | 4,145 | 0.129 | 5 | 2,802 | 324 | 1.0805 | 5,325 | 249 | 573 |
| R77Z | 9 | 2% | 31 | 9.53 | 47 | 4.416 | 17,667 | 0.098 |  | 0 | 353 | 1.168 | 8,238 | 165 | 518 |
| A36B | 8 | 2% | 44 | 21.64 | 46 | 7.095 | 28,385 | 0.224 |  | 0 | 505 | 2.1653 | 22,100 | 393 | 898 |
| A36A | 6 | 1% | 66 | 12.00 | 57 | 11.729 | 46,924 | 0.265 | 10 | 10,248 | 762 | 2.6035 | 39,322 | 524 | 1,287 |
| R61G | 6 | 1% | 23 | 7.60 | 16 | 0.992 | 3,969 | 0.125 | 8 | 3,917 | 105 | 0.7667 | 4,026 | 54 | 159 |
| A15C | 5 | 1% | 64 | 19.22 | 35 | 5.38 | 21,524 | 0.201 | 30 | 23,803 | 504 | 1.226 | 17,934 | 199 | 703 |
| Other* | 18 | 4% |  |  |  |  |  |  |  |  | 389 |  |  | 245 | 634 |
| **Total** | **450** | **100%** |  |  |  |  |  |  |  |  | **9,728** |  |  | **6,122** | **15,850** |

*Abbreviations: CAR-T – Chimeric Antigen Receptor T-Cell; DRG – diagnosis-related groups, LOS – length of stay; * Due to data protection reasons, case numbers less than four cases (“other”) are not disclosed in the InEK data browser.*

Supplementary Table 9: Cost Calculation for SCT administration (DRG-based)

| **DRG** | **Cases [n]** | **Cases [%]** | **Average LOS [days]** | **SD LOS [days]** | **First day with additional DRG costs [upper LOS]** | **Case mix average LOS [points]** | **DRG costs average LOS [€]** | **Case mix upper LOS [points]** | **Total days with additional DRG costs upper LOS [days]** | **DRG costs upper LOS [€]** | **Total DRG costs weighted [€]** | **Case mix nursing fee [points]** | **Nursing fees [€]** | **Nursing fees weighted [€]** | **Total costs weighted [€]** |
| --- | --- | --- | --- | --- | --- | --- | --- | --- | --- | --- | --- | --- | --- | --- | --- |
| **2020** |  |  |  |  |  |  |  |  |  |  |  |  |  |  |  |
| A15C | 584 | 96.37% | 27 | 11.30 | 36 | 6.19 | 22,725 | 0.23 |  | 0 | 21,900 | 1.2345 | 5,450 | 5,252 | 27,152 |
| A09A | 7 | 1.16% | 69 | 16.06 | 75 | 23.149 | 84,984 | 0.32 |  | 0 | 986 | 3.8824 | 44,685 | 518 | 1,504 |
| A11B | 7 | 1.16% | 69 | 15.19 | 56 | 14.627 | 53,698 | 0.27 | 14 | 13,791 | 783 | 3.4999 | 39,865 | 462 | 1,245 |
| Other* | 8 | 1.32% |  |  |  |  |  |  |  |  |  |  |  |  |  |
| **Total** | **606** | **100%** |  |  |  |  |  |  |  |  | **23,983** |  |  | **6,316** | **30,298** |
|  |  |  |  |  |  |  |  |  |  |  |  |  |  |  |  |
| **2021** |  |  |  |  |  |  |  |  |  |  |  |  |  |  |  |
| A15C | 560 | 96% | 27 | 11.23 | 35 | 6.078 | 22,780 | 0.23 |  | 0 | 21,882 | 1.2738 | 5,562 | 5,343 | 27,224 |
| A13A | 7 | 1% | 31 | 17.56 | 51 | 11.359 | 42,573 | 0.28 |  | 0 | 511 | 3.2804 | 16,738 | 201 | 712 |
| Other* | 16 | 3% |  |  |  |  |  |  |  |  |  |  |  |  |  |
| **Total** | **583** | **100%** |  |  |  |  |  |  |  |  | **23,025** |  |  | **5,700** | **28,725** |
|  |  |  |  |  |  |  |  |  |  |  |  |  |  |  |  |
| **2022** |  |  |  |  |  |  |  |  |  |  |  |  |  |  |  |
| A15C | 542 | 97% | 27 | 10.97 | 35 | 6.043 | 23,163 | 0.22 |  | 0 | 22,379 | 1.2432 | 5,981 | 5,779 | 28,158 |
| Other* | 19 | 3% |  |  |  |  |  |  |  |  |  |  |  |  |  |
| **Total** | **561** | **100%** |  |  |  |  |  |  |  |  | **23,163** |  |  | **5,981** | **29,145** |
|  |  |  |  |  |  |  |  |  |  |  |  |  |  |  |  |
| **2023** |  |  |  |  |  |  |  |  |  |  |  |  |  |  |  |
| A15C | 460 | 94% | 25.95 | 10.97 | 35 | 5.38 | 21,524 | 0.2 |  | 0 | 20,247 | 1.226 | 7,317 | 6,883 | 27,131 |
| A13A | 9 | 2% | 30.44 | 11.28 | 49 | 11.272 | 45,096 | 0.28 |  | 0 | 830 | 3.0363 | 21,258 | 391 | 1,221 |
| Other* | 20 | 4% |  |  |  |  |  |  |  |  |  |  |  |  |  |
| **Total** | **489** | **100%** |  |  |  |  |  |  |  |  | **21,976** |  |  | **7,585** | **29,561** |

*Abbreviations: DRG – diagnosis-related groups, LOS – length of stay, SCT – autologous stem cell therapy; * Due to data protection reasons, case numbers less than four cases (“other”) are not disclosed in the InEK data browser.*
